# Experimental Investigation of Steel-Borne Acoustic Pulses for Fault Pinpointing in Pipe-Type Cable Systems: A Scaled-Down Model Approach

**DOI:** 10.3390/s24217043

**Published:** 2024-10-31

**Authors:** Zaki Moutassem, Gang Li, Weidong Zhu

**Affiliations:** 1 Michael W. Hall School of Mechanical Engineering, Mississippi State University, Mississippi State, MS 39762, USA; zm336@msstate.edu; 2 Underground Systems Inc., Bethel, CT 06801, USA; 3 Department of Mechanical Engineering, University of Maryland, Baltimore County, Baltimore, MD 21250, USA; wzhu@umbc.edu

**Keywords:** acoustic pinpointing, fault pinpointing, fault location, HPFF, underground power transmission

## Abstract

Pipe-type cable systems, including high-pressure fluid-filled (HPFF) and high-pressure gas-filled cables, are widely used for underground high-voltage transmission. These systems consist of insulated conductor cables within steel pipes, filled with pressurized fluids or gases for insulation and cooling. Despite their reliability, faults can occur due to insulation degradation, thermal expansion, and environmental factors. As many circuits exceed their 40-year design life, efficient fault localization becomes crucial. Fault location involves prelocation and pinpointing. Therefore, a novel pinpointing approach for pipe-type cable systems is proposed, utilizing accelerometers mounted on a steel pipe to capture fault-induced acoustic signals and employing the time difference of arrival method to accurately pinpoint the location of the fault. The experimental investigations utilized a scaled-down HPFF pipe-type cable system setup, featuring a carbon steel pipe, high-frequency accelerometers, and both mechanical and capacitive discharge methods for generating acoustic pulses. The tests evaluated the propagation velocity, attenuation, and pinpointing accuracy with the pipe in various embedment conditions. The experimental results demonstrated accurate fault pinpointing in the centimeter range, even when the pipe was fully embedded, with the acoustic pulse velocities aligning closely with the theoretical values. These experimental investigation findings highlight the potential of this novel acoustic pinpointing technique to improve fault localization in underground systems, enhance grid reliability, and reduce outage duration. Further research is recommended to validate this approach in full-scale systems.

## 1. Introduction

Underground transmission systems are integral to modern power infrastructure, offering increased reliability and aesthetic benefits over overhead lines [1]. These systems primarily utilize three types of cables: cross-linked polyethylene (XLPE), self-contained fluid-filled (SCFF), and high-pressure fluid-filled (HPFF) or high-pressure gas-filled (HPGF) cables, each with unique characteristics and applications in power transmission [2]. The reliability of these systems hinges on effective fault location techniques and minimized outage durations. In the United States, over 80% of the 4200 circuit miles of underground high-voltage transmission cables are pipe-type cables, a significant portion of which have approached or exceeded their 40-year design life as of 2007 [3]. The aging infrastructure increases the risk of faults, underscoring the critical need for rapid and accurate fault location methods. A significant majority (69%) of faults occur in cable accessories rather than in the cables themselves, with installation mistakes accounting for 57% of accessory faults. For cable faults, production and installation errors are the primary causes (52%), followed by external damage (17%) and aging (9%) [4]. In HPFF cables, temperature-driven deterioration in oil-impregnated paper insulation is a significant issue. This deterioration approximately doubles in rate with every 6 °C increase in temperature and is exacerbated by oxygen and moisture ingress [5].

Faults in underground cables can take the form of open circuit faults, short circuit faults, or earth faults, each requiring appropriate identification and resolution [6]. These faults are inevitable and can lead to significant disruptions in power transmission and distribution. Prompt identification and resolution are essential for minimizing revenue losses and reducing customer inconvenience. The fault location process typically involves a two-step approach: prelocation followed by pinpointing. During prelocation, the cable circuit is tested from its terminations to estimate the distance to the fault. Effective prelocation can determine the fault’s location within a few percent of the total cable length; however, the accuracy may be reduced in very long cables. Some widely used prelocation techniques include time domain reflectometry (TDR), burn down, and arc reflection [7,8,9]. The choice of prelocation technique often depends on the fault type and cable characteristics, as no single method is universally optimal.

Pinpointing the exact fault location of underground cables can be accomplished through various techniques. Voltage gradient methods involve applying a pulsed direct current (DC) voltage to the faulty cable, creating a voltage gradient in the surrounding soil. This voltage gradient can be measured with earth probes to locate the fault, which is particularly effective for direct-buried cables but less accurate in highly resistive soils or ducts [10,11]. The magnetic gradient method detects the magnetic field from an alternating current (AC) voltage injected into the cable, useful for submarine cables but less effective in complex urban environments [12]. An acoustic method is the conventional approach for fault pinpointing and involves using “capacitive discharge” techniques [13]. This acoustic method involves discharging an electrical impulse onto the failed cable with a high-voltage test set, causing it to flash over at the fault’s location. The sound of the breakdown pulse is then picked up acoustically. This is commonly referred to as ‘thumping’. Pinpointing is accomplished by operators using specialized surface microphones (geophones) and walking along the cable route. While this method is very effective, it is burdened by an extremely slow response time due to its setup, the methodical search along the cable route, and interference or noise effects in the subsurface environment. Additionally, repeated thumping may cause further damage to the cable [14]. Locating faults in ducted cable systems can also be challenging. Poor acoustic contact may prevent the fault from generating a detectable signal at the surface, with the acoustic signal traveling along the duct and the strongest signal often detected at nearby manholes rather than directly at the fault site [15].

In recent years, newer fault location methods for underground cables, such as distributed temperature sensing and distributed vibration sensing, have shown promise in fault location [12]. These fault location methods utilize fiber-optic cables embedded alongside power cables to detect variations in temperature and vibrations caused by faults. While these fault location methods offer real-time monitoring and high precision, they require pre-installed fiber optics, which are not always available in legacy systems. Another growing trend is the integration of IoT-based systems, which combine technologies such as LTE, GPS, and GSM, along with electric field energy harvesters as power sources, to enable real-time fault detection and location [16,17,18,19,20,21,22,23]. These IoT-based fault location methods enable remote monitoring but are typically part of online systems that must be installed before a fault occurs, making them less practical for older circuits lacking such infrastructure. The limitations of current fault pinpointing methods, particularly for aging pipe-type cable systems in complex environments, highlight a critical gap in the field. While recent advancements focus on real-time, pre-installed systems, there is a pressing need for innovative offline solutions applicable to existing infrastructure. Current techniques, though offering certain advantages, fail to fully address the unique challenges posed by urban infrastructure and ducted systems. An ideal solution would leverage advanced sensors and technology for rapid, accurate, and non-damaging fault location, while building upon the strengths of acoustic detection methods.

To address these challenges, this study proposes a novel enhancement to conventional acoustic thumping for pipe-type cable systems. Rather than relying on the traditional method of walking the entire cable route with a geophone, the proposed pinpointing approach for pipe-type cable systems involves accessing nearby manholes and attaching acoustic sensors directly to the steel pipe after fault prelocation estimation. This innovative method, inspired by the concept of acoustic leak location as detailed in previous research [24,25,26,27,28], aims to offer a more efficient and accurate means of fault pinpointing. Hydrophones, geophones, and accelerometers were evaluated as candidate sensors for steel-borne acoustic pinpointing [29,30,31]. Among these, an accelerometer-based instrumentation package was identified as offering the best pinpointing accuracy, particularly for detecting acoustic pulses in steel pipes with low damping and a high signal-to-noise ratio [32]. Various signal processing techniques, such as Fast Fourier Transforms (FFTs), Wavelet Transforms (WTs), cross-correlation, and empirical mode decomposition (EMD), have been successfully applied to acoustic wave propagation in pipes [33,34,35,36,37,38]. While these methods can enhance data interpretation, this study focuses on validating direct accelerometer measurements for fault location. Future work may explore the integration of advanced signal processing techniques to further refine the pinpointing accuracy. By utilizing accelerometers in this enhanced setup, the proposed method aims to detect high-frequency steel-borne acoustic pulses with greater sensitivity and provide immediate and significantly improved fault pinpointing accuracy with just a single thump. Additionally, this approach minimizes the potential damage caused by repeated thumping inherent in conventional methods, which often involve manual searching with a geophone.

The main objective of this study is to validate the proposed concept of steel-borne acoustic fault pinpointing by replicating the thumping mechanism in the lab and carrying out an experimental study on a scaled-down HPFF test rig. This study will focus on determining the acoustic pulse propagation characteristics, evaluating the pinpointing accuracy, and assessing how the surrounding backfill material affects signal attenuation. The results will demonstrate the feasibility of this enhanced method in improving fault pinpointing for HPFF pipe-type cables. The successful implementation of this advanced acoustic pinpointing method could significantly improve the reliability and resilience of underground power distribution networks, potentially reducing downtime, minimizing repair costs, and enhancing the overall grid stability across urban and suburban areas.

The rest of this paper is organized as follows. Section 2 provides an overview of the theoretical foundations of acoustic pulse propagation, attenuation, and pinpointing concepts, offering essential context for the experimental methods discussed in subsequent sections. Section 3 details the experimental setup, including the scaled-down HPFF test pipe, instrumentation, and testing methodologies for acoustic pulse generation and detection. Section 4 presents the results, covering acoustic pulse velocity measurements, fault localization accuracy, and attenuation studies under various pipe embedment conditions. Section 5 discusses the implications of these results, primarily focusing on the potential of this technology for fault pinpointing and describing its advantages over the conventional method. Section 6 summarizes the conclusions drawn from this study. Finally, Section 7 suggests directions for future work.

## 2. Theoretical Background

Acoustic waves propagate along the wall of steel pipes as elastic deformations of the material. This wave motion can be described by the one-dimensional wave equation:

(1) $$\frac{\partial^{2}u}{\partial t^{2}}=v^{2}\frac{\partial^{2}u}{\partial x^{2}}$$

where *u* is the displacement, *t* is the time, *x* is the position along the length of pipe, and *v* is the velocity of the acoustic pulse. The theoretical velocity *v* of an acoustic pulse can be determined using the following equation:

(2) $$v=\sqrt{\frac{E}{\rho }}$$

where *E* is the Young’s modulus of the material and *ρ* is the density of the material. The general solution to Equation (1) for one-dimensional wave propagation is

(3) $$u\left(x,t\right)=f\left(x-vt\right)+g(x+vt)$$

where *u*(*x*,*t*) is the displacement at position *x* and time *t*, *v* is the wave velocity, *f*(*x* − *vt*) represents a wave traveling to the right, and *g*(*x + vt*) represents a wave traveling to the left.

As these waves propagate through the medium, they undergo attenuation due to several factors. Internal friction within the pipe material converts some of the wave energy into heat, leading to a gradual loss of amplitude. Additionally, when the pipe is embedded in soil, further attenuation occurs due to energy leakage into the surrounding environment. This interaction results in more complex wave behavior as energy is dissipated not only within the pipe but also at the interface with the soil. To account for this attenuation, we can modify Equation (3) to include an exponential decay factor, representing the attenuation coefficient *α*:

(4) $$u\left(x,t\right)=e^{-\alpha x}\left(f\left(x-vt\right)+g\left(x+vt\right)\right)$$

In this modified equation, *α* quantifies the rate of attenuation, indicating how quickly the wave amplitude diminishes as it propagates along the medium and into the surrounding environment. This formulation allows for a more accurate representation of wave behavior in practical scenarios, such as when acoustic pulses travel along a steel pipe embedded in soil.

In this study, the attenuation coefficient is determined experimentally, considering the complex interactions between the acoustic waves and the material properties of the pipe, as well as the characteristics of the surrounding medium, such as soil. Theoretical models often oversimplify these interactions, failing to account for variables such as variations in soil density, moisture content, and the mechanical properties of the pipe material.

As a result, relying solely on theoretical relationships can lead to inaccuracies in predicting wave behavior and attenuation rates. By conducting experimental studies, we can capture the real-world dynamics at play, allowing us to obtain a more reliable attenuation coefficient that reflects the actual performance of the acoustic system in situ. This experimental approach not only enhances the validity of our findings but also provides a foundation for future studies aimed at optimizing acoustic fault pinpointing techniques in various environments. Experimentally, the attenuation coefficient α is calculated using the following equation:

(5) $$\alpha =-\frac{\ln\left(A_{1}/A_{0}\right)}{L}$$

where *A*_0_ is the reference amplitude and *A*_1_ is the amplitude of the acoustic pulse after traveling a distance *L*.

Thumping a faulted cable generates an acoustic pulse at the fault location that travels along the pipe in both directions, as described by Equation (4), potentially captured by time-synchronized sensors positioned at each manhole. Due to the varying distances the acoustic pulse travels, the difference in the arrival times at each sensor can be represented as

(6) $$∆t=t_{2}-t_{1}$$

To pinpoint the source of the acoustic pulse, we used a time difference of arrival (TDOA) method. The TDOA method can be expressed mathematically as

(7) $$d_{1}=\frac{d-v∆t}{2}$$

where *d* is the total length of the pipe and ∆*t* is the previously defined time difference of arrival.

## 3. Experimental Setup: Materials and Methods

**Figure 1:**
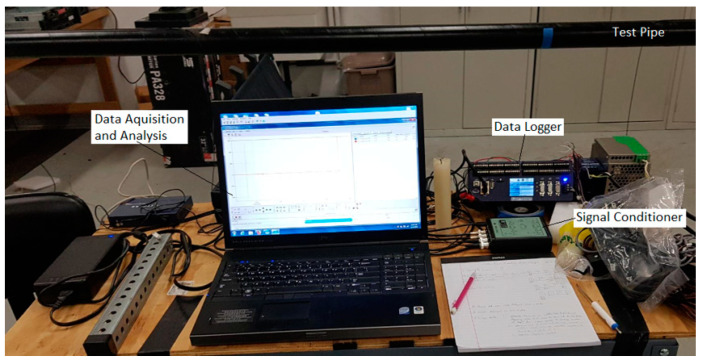
Test pipe and measurement system components.

Acoustic pinpointing feasibility testing was conducted on an experimental test pipe consisting of a 19 m long, Schedule-80 carbon steel pipe, (49.25 mm Inner Diameter), the same material used in HPFF pipes. The pipe was coated externally with Pritec (HDPE over butyl mastic; Liberty Coating Company, LLC, Hatfield, PA, USA), the same coating used on HPFF pipes. The pipe’s dimensions and material properties are summarized in Table 1. The measurement system included four B&K accelerometers (Brüel & Kjær North America Inc., Duluth, GA, USA), two B&K two-channel signal conditioners, and one Delphin Expert Transient Data Logger (Delphin Technology AG, Bergisch Gladbach, Germany), with specifications provided in Table 2, Table 3 and Table 4. Figure 1 depicts the test pipe and the components of the measurement system.

**Figure 2:**
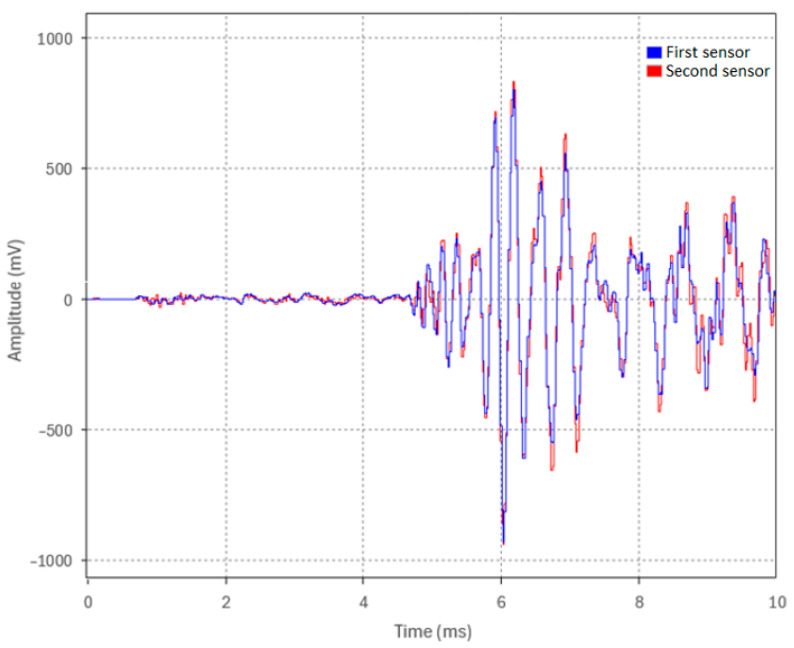
Calibration and time synchronization of two accelerometers.

The B&K accelerometers were individually calibrated by the manufacturer using state-of-the-art random FFT technology, providing an 800-point high-resolution calibration (magnitude and phase). To further verify both calibration and time synchronization, the sensors were mounted together at one end of the pipe while an acoustic pulse was generated by an impact at the opposite end. Since both sensors were at the same location, they should measure an identical pulse timing and amplitude. Figure 2 shows representative waveforms of the acoustic pulse from the first and second sensors, demonstrating their high degree of time synchronization and calibration accuracy in relation to each other. The same verification procedure was performed for the remaining two sensors.

**Figure 3:**
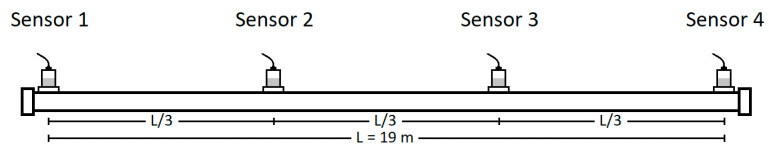
Sensor placements on test pipe (Sensors 1–4).

Following verification, the sensors were attached using beeswax to the steel pipe at strategic locations: both ends and at one-third intervals along its length. These positions, referred to as Locations 1, 2, 3, and 4 in the text, correspond to Sensors 1, 2, 3, and 4 shown in Figure 3. This configuration enabled comprehensive data collection along the entire pipe length.

Two methods were employed for acoustic pulse generation in the test pipe for this study. The first method utilized a weight drop apparatus where a 0.91 kg (2 lb) weight was released from a fixed height of 63.5 mm (2.5 in) onto the pipe’s surface. This method ensured consistent and repeatable acoustic pulse generation, enabling standardized measurements across multiple tests and runs.

The second method employed a fault simulator device designed to more closely mimic the real-world fault thumping mechanism. This custom-built device comprised a non-resistor spark plug, retrofitted into the dielectric fluid filled as will be detailed in Section 3.2. The terminals of the fault simulator device connect to a surge wave generator or a full-scale thumper, enabling powerful capacitive discharges at the spark gap. The specifications of the full-scale thumper are detailed in Table 5.

### 3.1. Acoustic Pulse Velocity and Pinpointing Accuracy Test Procedures (Test Series 1)

#### 3.1.1. Determining and Verifying Acoustic Pulse Velocity in Steel Pipe (Test 1.1)

To accurately pinpoint the source of an acoustic pulse along the length of the pipe, it is essential to first determine the acoustic pulse velocity in the steel pipe experimentally and validate it against the theoretical value. In Test 1.1, an acoustic pulse was generated at Location 1 using the weight drop apparatus. The time of flight across the 19 m test pipe was measured by cross-correlating the pulse’s arrival time at Location 4 with its initiation time at Location 1. The experimental velocity of the acoustic pulse was then calculated using the distance and measured time, while the theoretical velocity of the acoustic pulse was calculated using Equation (2).

#### 3.1.2. Localization Accuracy and Pinpointing of Acoustic Pulse Source (Test 1.2)

With the propagation velocity established, Test 1.2 aimed to simulate a real-world fault thumping scenario by evaluating the ability to pinpoint an acoustic source using the TDOA method described by Equation (7). In actual field conditions, cable faults could occur at any point along the pipe. To simulate this, Location 2, corresponding to one-third of the 19 m test pipe length, was arbitrarily selected and an acoustic pulse was generated there using the weight drop apparatus. The test assessed whether sensors positioned at the pipe ends could be used to accurately pinpoint the source location, with the accuracy expressed as a percentage of the pipe length, thus validating the fault pinpointing concept.

### 3.2. Fault Simulator Device Evaluation for Acoustic Pulse Generation Test Procedure (Test 2)

**Figure 4:**
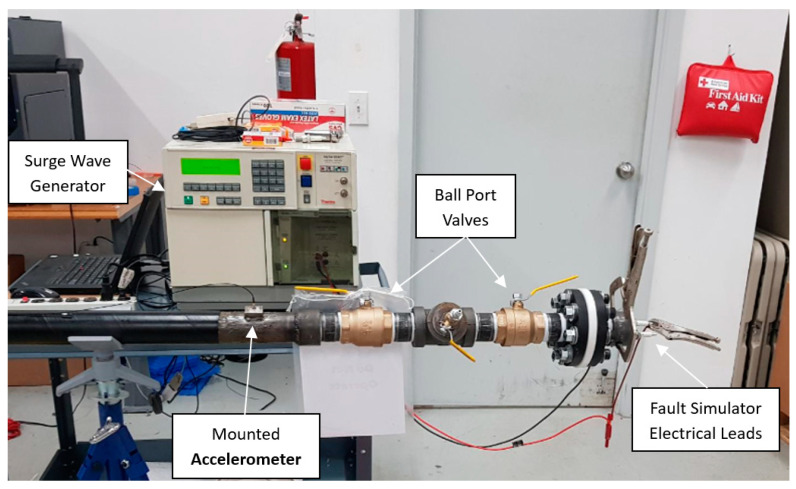
Fault simulator device connected to the surge wave generator.

For the proposed technology to be viable for HPFF fault pinpointing, the thumping of the cable system must vaporize the dielectric fluid surrounding the fault. The sudden vaporization of the dielectric fluid creates a shock wave, which is believed to induce an acoustic pulse that propagates inside the pipe’s wall in both directions. To investigate this concept, the test pipe was equipped with the necessary hydraulic components and filled with dielectric fluid type DF 100, a fluid very commonly used in HPFF cable systems. The fault simulator device was then installed in the test pipe and connected to the surge wave generator, as shown in Figure 4. Test 2 was conducted to validate the concept that thumping a fault generates an acoustic pulse in the steel wall. This test involved producing a capacitive discharge (spark) in the dielectric fluid at Location 1. The goal was to generate a steel-borne acoustic pulse that could be detected by Sensor 4 at the remote end of the pipe. This test laid the groundwork for more comprehensive future testing.

### 3.3. Acoustic Pulse Attenuation Evaluation Testing Procedures (Test Series 3)

In actual HPFF pipe-type cable installations, the steel pipe is mechanically coupled to the surrounding soil, resulting in increased signal attenuation due to the leakage of acoustic energy into the soil. To explore this complex interaction, Test 3 was conducted as a series of subtests (Tests 3.1–3.3), designed to investigate the effects of varying embedding conditions on the propagation and attenuation of steel-borne acoustic pulses. The tests included conditions where the pipe was fully supported in air (Test 3.1), laid on tamped rock dust (Test 3.2), and, finally, fully embedded in tamped rock dust (Test 3.3). Rock dust, selected for its excellent thermal properties and common use in HPFF installations, served as the backfill. Its grain composition, which ranges from coarse to fine, allows for high compaction and thermal conductivity, providing a controlled medium to analyze acoustic energy absorption and signal attenuation.

In each subtest (Tests 3.1–3.3), a consistent acoustic pulse was generated using the weight drop apparatus, with a 0.91 kg (2 lb.) weight dropped from a constant height of 63.5 mm (2.5 in) at the pipe end (Location 1). The four accelerometers were evenly spaced along the 19 m pipe at one-third intervals (6.33 m apart). To ensure reliability and repeatability, five identical runs were recorded for each subtest. A 3 kHz low-pass filter (LPF) was applied to the data to remove high-frequency noise and enhance the clarity of the acoustic pulse signals. This filter was used because it was observed in previous tests that the acoustic pulse frequency components resided within this range. The filtered data were then collected and analyzed to assess the impact of different embedding conditions on signal attenuation. The attenuation coefficient between sensors was calculated using Equation (5).

#### 3.3.1. Pipe Supported in Air—Attenuation of Steel Pipe (Test 3.1)

**Figure 5:**
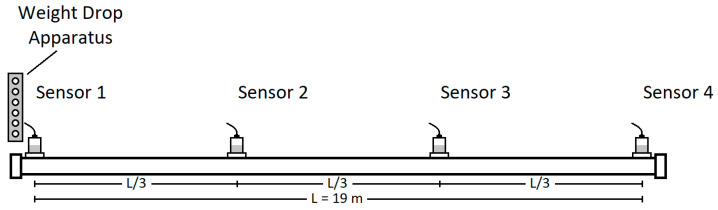
Pipe supported in air—Test 3.1 configuration.

In Test 3.1, the test pipe was supported in air using four V-head pipe stands, identical to the setup in Test Series 1 and 2. This test aimed to assess the attenuation of the acoustic pulse solely due to the pipe’s properties, serving as a baseline reference for evaluating the additional damping effects observed in Tests 3.2 and 3.3. Figure 5 depicts the setup for Test 3.1, showing the pipe supported in air along with the location of the weight drop apparatus.

#### 3.3.2. Pipe Laid on Tamped Backfill—Attenuation Due to Contact with Backfill (Test 3.2)

**Figure 6:**
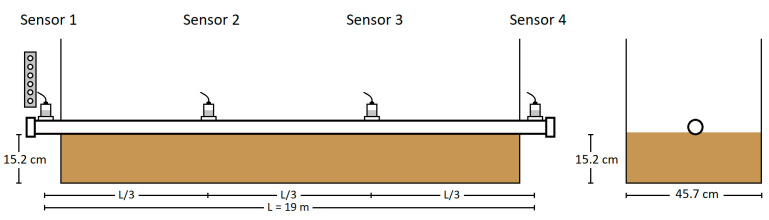
Test 3.2 configuration (pipe laid on tamped rock dust)—impact at Sensor 1 location.

**Figure 7:**
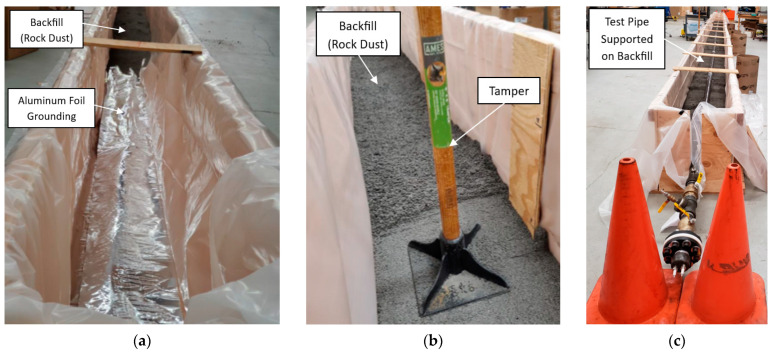
Tamping and layering process: (**a**) rock dust being poured into the containment box; (**b**) rock dust tamped in 5–8 cm lifts; and (**c**) final setup of the pipe laid on 15 cm of tamped rock dust in Test 3.2.

For Test 3.2, a containment box measuring 17 m in length, 46 cm in width, and 61 cm in height was constructed from plywood and lined with a vinyl sheet to prevent moisture ingress. Aluminum foil was placed at the bottom of the box to provide grounding for future thumping tests. The pipe was placed on top of the 15 cm thick layer of tamped rock dust, which was added and tamped in 5–8 cm lifts. The overall test layout with the dimensions of the tamped rock dust is shown in Figure 6. The tamping and layering process are depicted in Figure 7a,b, and the final setup is shown in Figure 7c. As in Test 3.1, controlled weight drops were performed using the weight drop apparatus from the same height of 63.5 mm (2.5 in), keeping all parameters consistent except for the pipe now being supported on the tamped rock dust.

#### 3.3.3. Attenuation in Fully Embedded Pipe (Test 3.3)

**Figure 8:**
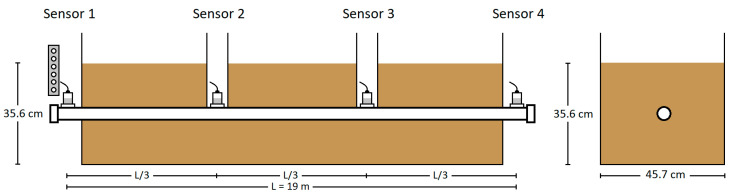
Test 3.3 configuration (fully embedded)—impact at Sensor 1 location.

**Figure 9:**
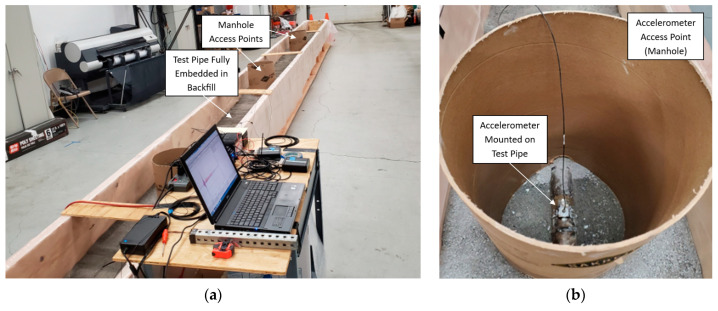
Test 3.3—fully embedded configuration: (**a**) complete experimental setup and (**b**) manhole access points at intermediate locations for accelerometer placement.

In Test 3.3, additional layers of rock dust were added and tamped until the pipe was fully embedded in the center of the containment box, as shown in Figure 8 and Figure 9a. Access to the intermediate sensors via a ‘manhole’ is shown in Figure 9b. The fully surrounding backfill material (rock dust) provided an absorbing medium for acoustic energy, similar to real pipe-type cable systems. Controlled weight drops were performed using the weight drop apparatus, with all the parameters consistent with those in Tests 3.1 and 3.2, except that the pipe was now fully embedded in the backfill.

### 3.4. Fully Embedded Fault Simulator Pinpointing Test Procedure (Test 4)

**Figure 10:**
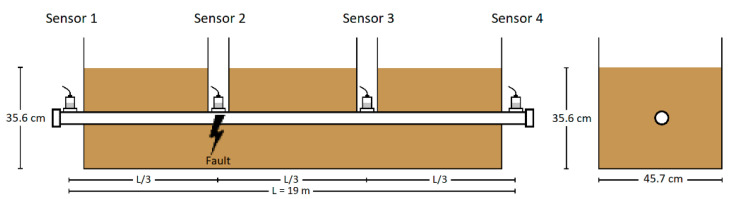
Test 4 configuration (fully embedded)—fault simulated at Sensor 2 location.

Test 4 evaluated the acoustic pinpointing method in the fully embedded, dielectric fluid-filled configuration of Test 3.3. The fault simulator was positioned at one-third of the pipe’s length (Location 2), as shown in Figure 10, to replicate the common occurrence of faults at an intermediate location in real systems. A full-scale commercial thumper applied a 16 kV voltage charge to generate arcing at the fault. The objective was to assess the acoustic pinpointing effectiveness under conditions simulating an actual HPFF system in a laboratory environment.

### 3.5. Summary of Experimental Tests

Table 6 summarizes all the tests conducted in this study, detailing the objectives, acoustic pulse generation methods, and corresponding embedment conditions for each test case.

## 4. Experimental Results

### 4.1. Acoustic Pulse Velocity and Pinpointing Accuracy Test Results (Test Series 1)

#### 4.1.1. Determining and Verifying Acoustic Pulse Velocity in the Test Pipe (Test 1.1)

**Figure 11:**
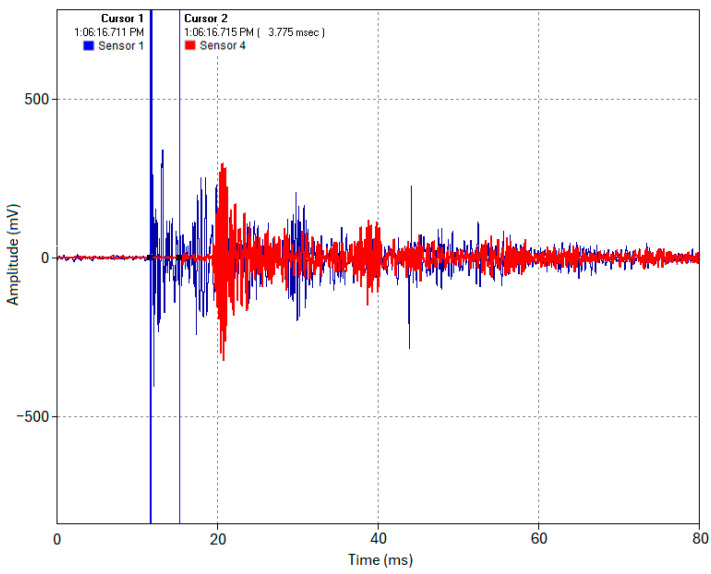
Acoustic waveforms captured by Sensors 1 and 4 (Test 1.1).

**Figure 12:**
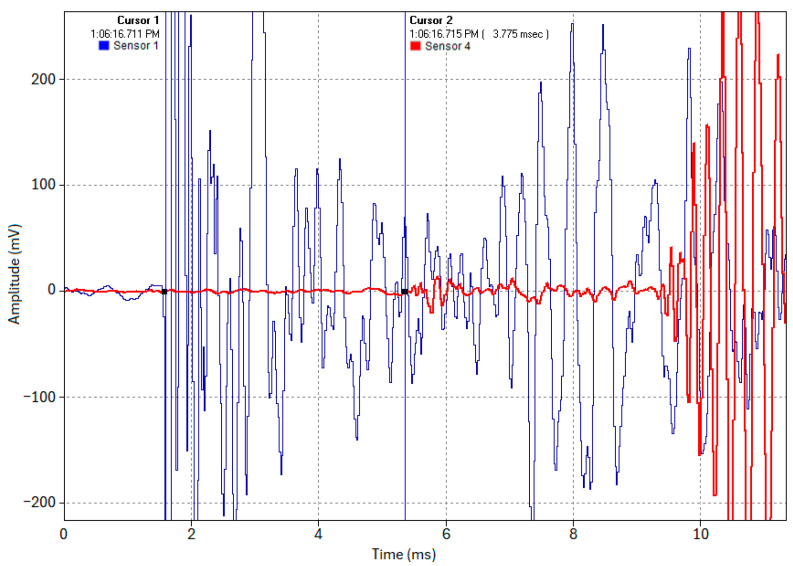
Close-up snapshot of the timestamping of the acoustic waveforms captured by Sensors 1 and 4 (Test 1.1).

In Test 1.1, an acoustic pulse was induced at the pipe’s end (Location 1). The sensors at Locations 1 and 4 captured the complete waveforms of this acoustic event, as shown in Figure 11. Cursor 1 indicates the timestamp of when the acoustic pulse was induced at Location 1 (Sensor 1, blue trace), and Cursor 2 indicates the timestamp of the time of arrival of the acoustic pulse at Location 4 (Sensor 4, red trace). The acoustic pulse’s time of flight across the 19 m pipe was determined using Equation (6) to be approximately 3.75–3.8 ms, as shown in the cross-correlated close-up snapshot in Figure 12. This yields an experimental propagation velocity of 5000–5066.67 m/s, closely matching the theoretical velocity of 5047.7 m/s (calculated using *E* = 200 GPa and *ρ* = 7850 kg/m^3^ in Equation (2)). Multiple test runs were conducted, and all yielded the same results with a max difference of about 0.1 ms between runs. The slight discrepancy between the test runs can be attributed to high-frequency wave timestamping limitations. While advanced signal processing might reduce this error, the current accuracy is sufficient for this study’s proof of concept purpose.

#### 4.1.2. Localization Accuracy When Pinpointing the Source of an Acoustic Pulse (Test 1.2)

**Figure 13:**
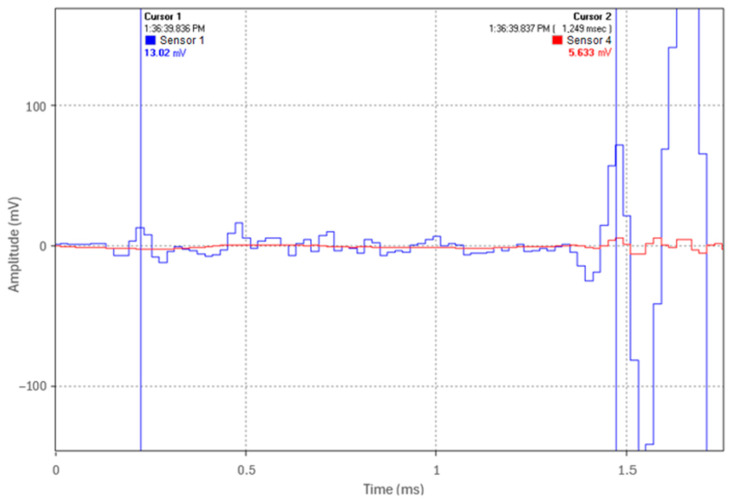
TDOA of the acoustic pulse at pipe ends when an acoustic pulse is induced at one-third the length of the pipe (Test 1.2).

In Test 1.2, to evaluate the viability and accuracy of the acoustic pinpointing concept, an acoustic pulse was induced at one-third of the pipe’s length (Location 2). Figure 13 illustrates the TDOA of the acoustic pulse at the pipe ends, which was measured as 1.249 ms. In Figure 13, Cursor 1 indicates the arrival time at Location 1 (Sensor 1, blue trace), while Cursor 2 indicates the arrival time at Location 4 (Sensor 4, red trace).

Using Equation (7), with ∆*t* = 1.249 ms, *v* = 5047.7 m/s, and *d* = 19 m, the acoustic pulse’s origin was calculated to be 6.348 m from Location 1. This closely matches the actual source location of 6.333 m (one-third of the pipe length). The deviation of 1.44 cm, corresponding to only 0.076% of the total pipe length, demonstrates the centimeter-level accuracy achieved in this test. This suggests that if cable thumping generates a sufficiently strong acoustic pulse that propagates along the steel pipe wall and can be detected by remotely positioned sensors, precise fault pinpointing could be achievable.

### 4.2. Fault Simulator Device Evaluation for Acoustic Pulse Generation Test Results (Test 2)

**Figure 14:**
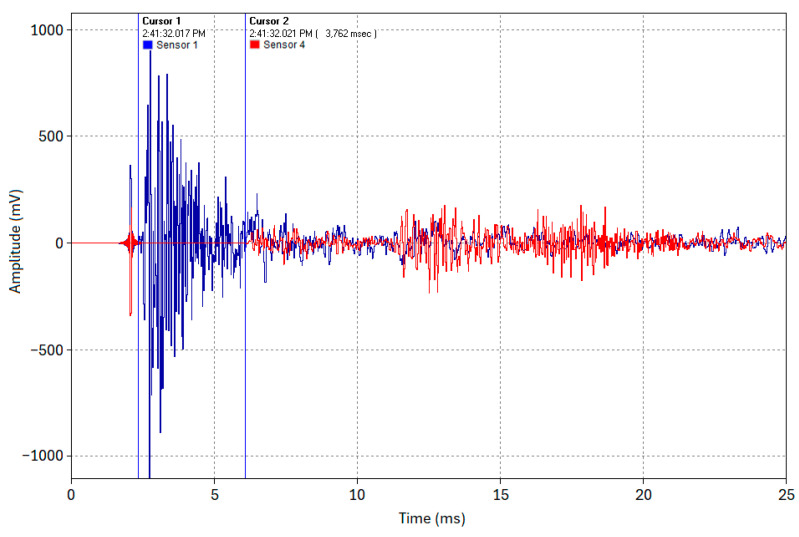
Test 2—blue waveform (Sensor 1); red waveform (Sensor 4).

Test 2 involved creating an arc (spark) in the dielectric fluid inside the pipe at Location 1, simulating the arcing mechanism that occurs at the fault location when a cable is thumped. The waveforms recorded by the sensors installed at the pipe ends are shown in Figure 14. The results demonstrate that a capacitive discharge in the dielectric fluid not only creates a hydraulic pressure transient but also induces an acoustic pulse, which propagates along the steel pipe wall. This test further validates the acoustic fault pinpointing concept, confirming that thumping a real HPFF pipe-type cable system will very likely induce an acoustic pulse at the fault’s location. The acoustic pulse time of flight across the 19 m pipe was 3.76 ms, consistent with that derived in Test 1.1. This consistency was expected since the acoustic velocity depends on the propagation medium, regardless of the generation method.

### 4.3. Acoustic Pulse Attenuation Evaluation Testing Results (Test Series 3)

#### 4.3.1. Testing Results of Pipe Supported in Air—Attenuation of Steel Pipe (Test 3.1)

**Figure 15:**
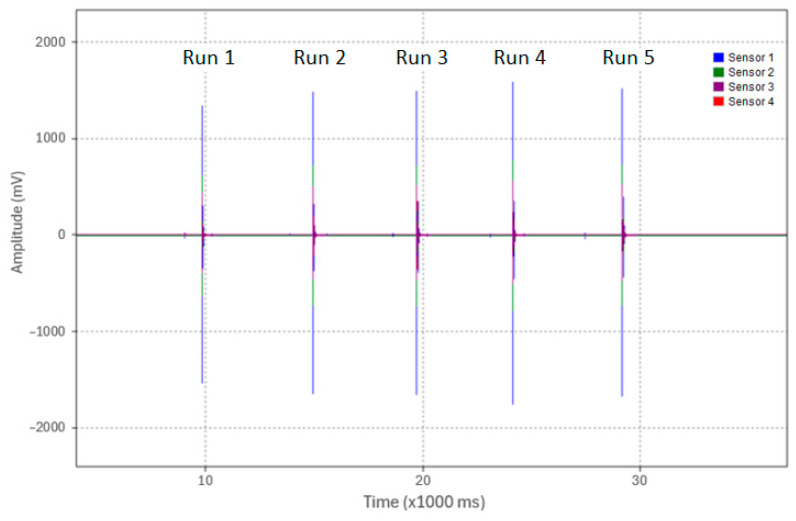
Overview of peak amplitudes from Sensor 1 for the five test runs of Test 3.1.

**Figure 16:**
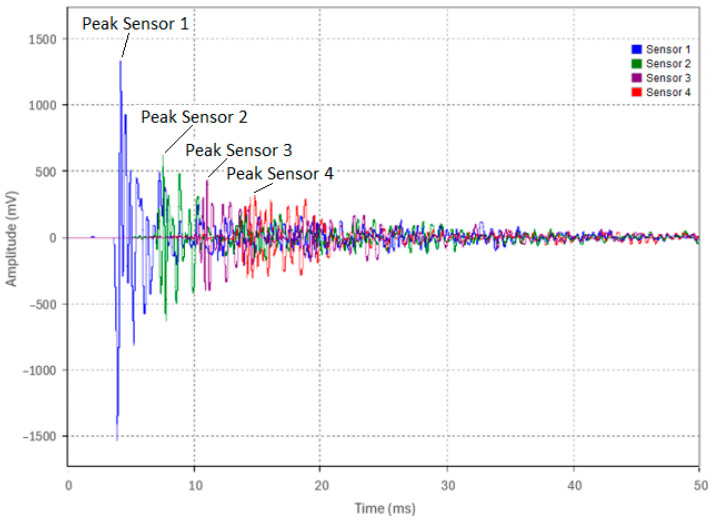
Captured waveforms from Sensors 1, 2, 3, and 4 for Run 1 in Test 3.1.

In Test 3.1, the pipe was supported in air similar to in the previous tests. An acoustic pulse was induced at the pipe end (Location 1) via the weight drop apparatus (0.91 kg dropped from a 63.5 mm height). Five identical test runs were conducted from the same height with a 3 kHz LPF applied to the experimental data. An overview of the five test runs is shown in Figure 15 and the waveforms of Test 1—Run 1 as acquired by the four sensors are shown in Figure 16. The waveform peak amplitudes from Sensors 1, 2, 3, and 4, as labeled in Figure 16, were recorded for all five runs and were tabulated in Table 7.

The attenuation of the acoustic pulse is evident, as shown in Figure 16 and summarized in Table 7. Table 7 also shows that the peak amplitudes between runs were consistent, confirming the reliability of the excitation method and the repeatability of the test setup. Using Equation (5), the attenuation coefficient between each sensor pair was calculated in Np/m and is presented in Table 7. The peak amplitude decreased by approximately 52% from Sensor 1 to Sensor 2, 29% from Sensor 2 to Sensor 3, and 31% from Sensor 3 to Sensor 4. This pattern of attenuation was consistent across all five test runs.

**Figure 17:**
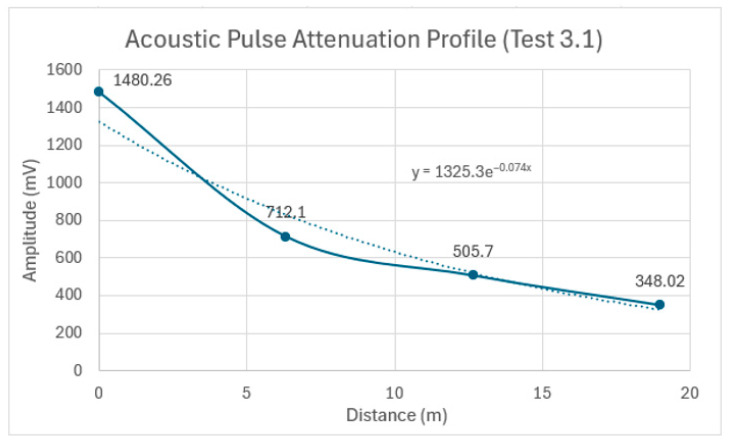
Acoustic pulse attenuation profile as it propagates along the 19 m pipe in Test 3.1: the solid line connects the data points, and the dotted line shows an exponential trendline that best fits the overall attenuation.

To determine the overall attenuation over the entire 19 m pipe, the average peak amplitude from each of the four sensors was plotted in Figure 17. This figure illustrates how the signal’s peak value attenuates as it propagates along the length of the pipe. The overall attenuation coefficient, determined to be 0.074 Np/m from the power of the exponential function in the trendline equation, will serve as a reference for assessing the added attenuation due to pipe embedment in the subsequent tests.

#### 4.3.2. Testing Results of Pipe Laid on Tamped Backfill—Attenuation Due to Contact with Backfill (Test 3.2)

**Figure 18:**
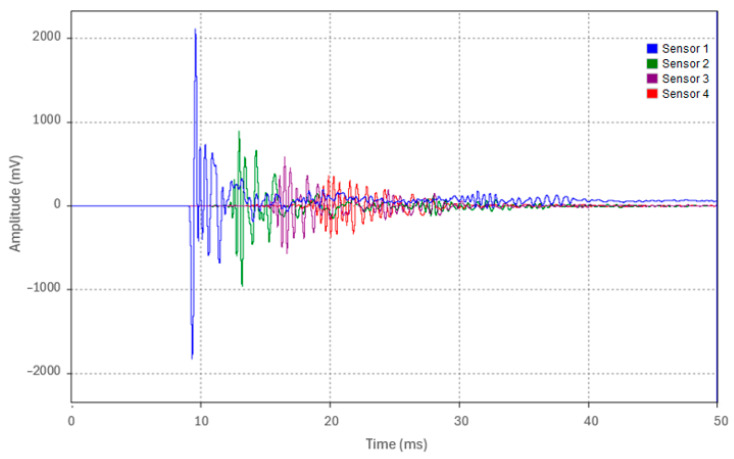
Waveforms from the four accelerometers of Run 1 in Test 3.2.

Test 3.2 replicated the procedure of Test 3.1, with the only modification being that the test pipe was placed on tamped backfill instead of being supported in air. The waveforms acquired from the four sensors for a representative run (Run 1) are shown in Figure 18. The acoustic pulse peak amplitudes at each sensor location for the five test runs are detailed in Table 8.

**Figure 19:**
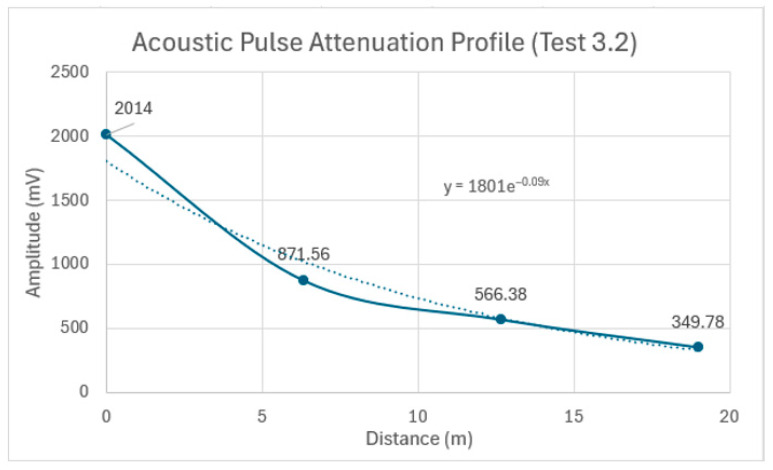
Acoustic pulse attenuation profile as it propagates along the 19 m pipe in Test 3.2: the solid line connects the data points, and the dotted line shows an exponential trendline that best fits the overall attenuation.

The attenuation of the acoustic pulse is evident, as shown in Figure 18 and summarized in Table 8. The percentage decrease in the peak amplitude was approximately 56.7% from Sensor 1 to Sensor 2, 35% from Sensor 2 to Sensor 3, and 38.2% from Sensor 3 to Sensor 4. Figure 19 depicts the overall attenuation of the acoustic pulse as it propagates along the 19 m pipe. The overall attenuation coefficient for Test 3.2 was measured at 0.09 Np/m. A slight increase in the attenuation coefficient of approximately 0.016 Np/m was observed between Tests 3.1 and 3.2. This increase is attributed to the pipe’s contact with tamped backfill, which resulted in minor acoustic energy leakage.

#### 4.3.3. Testing Results of Attenuation in Fully Embedded Pipe (Test 3.3)

**Figure 20:**
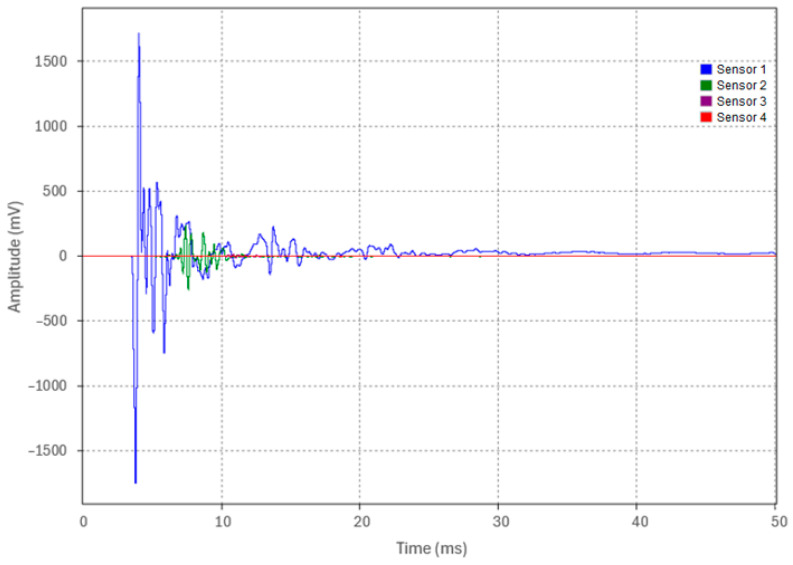
Waveforms from the four accelerometers of Run 1 in Test 3.3.

Test 3.3 replicated the testing procedures of Tests 3.1 and 3.2, with the exception that the pipe was fully embedded in backfill. The waveforms acquired from the four sensors for a representative run (Run 1) are shown in Figure 20. The acoustic pulse peak amplitudes at each location for the five test runs are detailed in Table 9.

**Figure 21:**
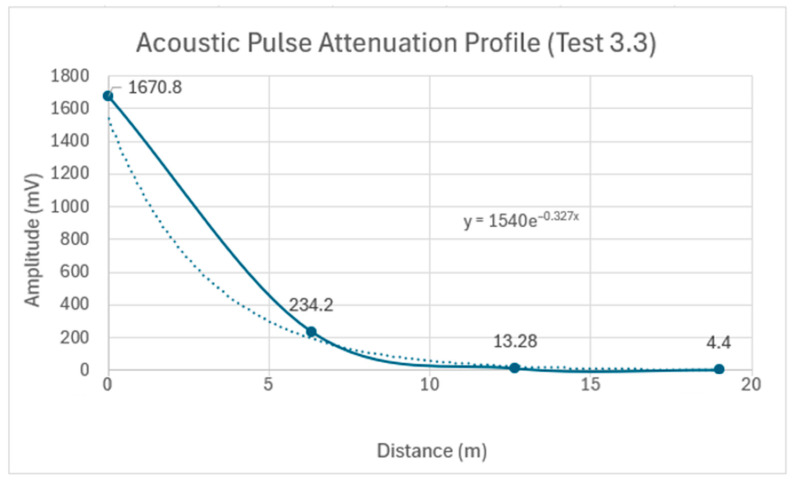
Acoustic pulse attenuation profile as it propagates along the 19 m pipe in Test 3.3: the solid line connects the data points, and the dotted line shows an exponential trendline that best fits the overall attenuation.

An analysis of the Test 3.3 data in Table 9 indicates that the percentage decrease in the peak amplitude was approximately 86% from Sensor 1 to Sensor 2, increasing to 94.3% from Sensor 2 to Sensor 3, and measuring at 66.9% from Sensor 3 to Sensor 4. The overall average attenuation across the 19 m embedded pipe for Test 3.3 was calculated to be 0.327 Np/m, as shown in Figure 21. An increase in the attenuation coefficient of approximately 0.237 Np/m was observed between Tests 3.2 and 3.3. This increase is attributed to the transition from the partial backfill contact at the pipe’s bottom surface in Test 3.2 to complete backfill embedment around the pipe’s circumference in Test 3.3, resulting in significant acoustic energy leakage into the surrounding medium. The data demonstrate that the acoustic pulse amplitude decreased from an initial 1670 mV to 4.7 mV after propagating the entire 19 m pipe length. However, despite this substantial reduction, the pulse remained clearly detectable after traveling 19 m, highlighting the potential viability of this method for long-distance acoustic pulse detection in fully embedded pipe conditions.

### 4.4. Fully Embedded Fault Simulator Pinpointing Test Results (Test 4)

**Figure 22:**
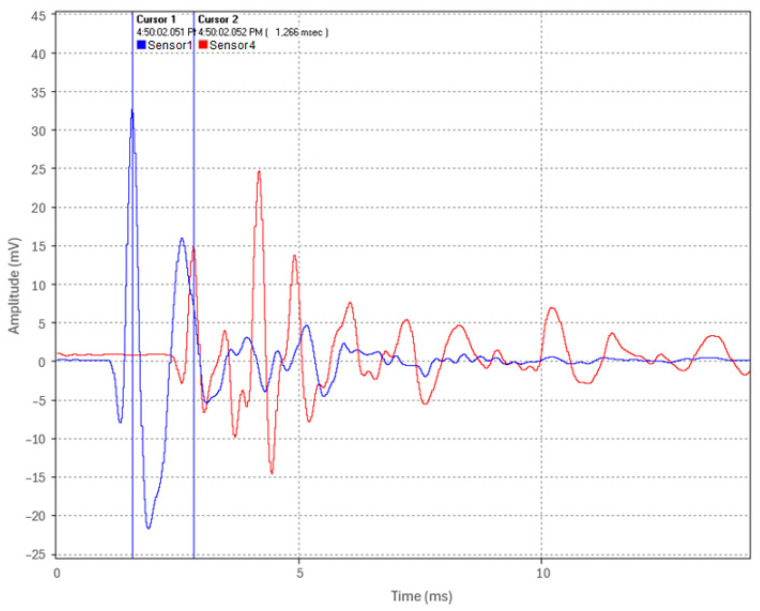
Fault-induced acoustic pulse waveform captured by Sensors 1 and 4 (Test 4).

Following the assessment of the backfill material’s impact on acoustic pulse attenuation, we confirmed that a low-amplitude pulse generated by the weight drop apparatus remained detectable after propagating 19 m in the fully embedded pipe. Subsequently, a test was conducted using a fault simulator and a full-scale commercial thumper, which applied a 16 kV voltage charge to simulate a fault thumping event at one-third of the pipe’s length (Location 2). Figure 22 shows the acoustic pulse waveform recorded by the time-synchronized sensors at the pipe’s ends (Sensor 1 in blue and Sensor 4 in red).

The results of the fault simulator test on the fully embedded pipe indicate that even when the test pipe is fully embedded in backfill, the instrumentation package can readily detect and timestamp the acoustic pulse waveform generated at the fault’s location using sensors positioned remotely (Figure 22). A time of arrival difference of 1.266 ms was deduced between Sensors 1 and 4. Using Equation (2), with ∆*t* = 1.266 ms, *v* = 5047.7 m/s, and *d* = 19 m, the fault-induced acoustic pulse origin was calculated to be 6.305 m from the pipe’s end (Location 1). This result closely aligns with the actual fault location at 6.333 m (one-third of the pipe length).

These findings indicate that the fault pinpointing in the scaled-down HPFF test setup was accurate to within 2.8 cm, representing an accuracy of within 0.15% of the pipe’s 19 m length. This demonstrates the potential of acoustic fault pinpointing for HPFF pipe-type cable systems, suggesting that high precision may be achieved within a typical test setup. However, the validation of a full-scale system is necessary.

## 5. Discussion

The studies presented in this paper provide significant insights into the feasibility and accuracy of a novel acoustic fault pinpointing technique within a scaled-down model of an HPFF pipe-type cable system. Utilizing a 19 m test pipe, our experiments demonstrated high accuracy in both measuring acoustic pulse velocity and localizing faults under controlled conditions. The measured velocity, ranging from 5000 to 5066.67 m/s, closely aligned with the theoretical value of 5047.7 m/s, thus validating the measurement methodology used in this study. Additionally, fault simulation tests conducted on the fully embedded configuration confirmed the potential applicability of this technique. The acoustic pulse generated by a commercial thumper propagated bidirectionally from the fault location, as described by Equation (4). The attenuation term in Equation (4) was evident as the pulse detected by the sensor farther from the source showed a greater amplitude reduction due to the longer travel distance. By utilizing the time difference of arrival between the sensors, we successfully detected and accurately localized a simulated fault to within 2.8 cm over the 19 m pipe. This precision corresponds to an accuracy of within 0.15% of the total pipe length, highlighting the effectiveness of this method for potential fault pinpointing in full-scale HPFF pipe-type cable systems.

The effect of the pipe embedment on the acoustic pulse attenuation was clearly demonstrated in Test Series 3. The attenuation progressively increased as we moved from the pipe suspended in the air to the partially and fully embedded conditions. Specifically, an attenuation coefficient of 0.074 Np/m was measured between Sensors 1 and 4 in the air-supported setup. This value increased to 0.09 Np/m when the pipe was laid on tamped backfill, and further to 0.327 Np/m when the pipe was fully embedded in tamped backfill. These results highlight the significant role that the surrounding material plays in acoustic energy dissipation.

Our analysis revealed a frequency spectrum within the range of 3 kHz during the fault simulation test. We expect that in real-world HPFF cable systems, the frequency of the acoustic pulse generated during thumping would be lower. This expectation arises from the increased arcing power and the larger cross-sectional area occupied by the dielectric fluid in the pipe, which may transmit a broader range of frequencies to the steel pipe wall. A lower-frequency pulse is likely to experience reduced attenuation, enabling fault pinpointing over longer distances. However, there may be a slight trade-off in the pinpointing accuracy due to the broader waveform associated with lower frequencies, which may result in less distinct timestamps compared to the sharp peaks of higher frequencies. Nonetheless, this reduction is not expected to hinder effective pinpointing.

The promising results presented here provide an encouraging step toward understanding these phenomena, but further empirical validation is required to confirm these assumptions in full-scale systems. Additional studies are needed to explore the detection range, accuracy, and reliability of this method under diverse environmental and operational factors present in an actual installation.

## 6. Conclusions

This study successfully demonstrated the feasibility and accuracy of a novel acoustic fault pinpointing method applied to a scaled-down model of HPFF pipe-type cable systems. The measurement of the acoustic pulse velocities, which closely aligned with the theoretical values, was essential for establishing the groundwork for accurate fault localization. The ability to detect and localize a fault to within 0.15% of the pipe’s total length using a commercial thumper suggests that the method has practical applications. While these findings are promising, they represent an initial step. Further confirmation using a full-scale HPFF system is essential to assess the method’s performance under real-world conditions. Specifically, the validation of the detection range, accuracy, and reliability will be critical in determining the technique’s practical utility. The observed effect of pipe embedment on the acoustic attenuation is another crucial finding. As demonstrated, the attenuation increased as the pipe transitioned from being suspended in air to fully embedded. This underscores the importance of understanding how surrounding materials affect acoustic signal propagation along the pipe and energy dissipation in real-world scenarios. In full-scale HPFF cable system acoustic pinpointing, we anticipate that lower-frequency pulses generated during thumping could result in reduced attenuation, thereby facilitating fault pinpointing over longer distances.

In conclusion, the successful implementation of this enhanced acoustic pinpointing method could significantly improve the reliability and resilience of underground power distribution networks. By addressing the limitations of traditional acoustic pinpointing techniques, which often suffer from slow response times due to extensive setup and methodical searches, as well as issues with noise interference and potential cable damage from repeated thumping, the proposed method offers a potentially faster and more accurate solution for fault pinpointing. Additionally, the proposed approach overcomes challenges faced in ducted systems, where poor acoustic contact can lead to detection at manholes rather than at the fault site. The use of advanced sensors in our scaled-down model demonstrates promising results that could reduce downtime, minimize repair costs, and enhance the overall grid stability in urban and suburban areas. The experimental findings in this study provide a solid foundation for full-scale testing and offer insights into acoustic pulse behavior and attenuation patterns that can guide future research and deployment strategies.

## 7. Future Work

To address the limitations identified in this study and further advance the research, we propose the following future work.

1. Field demonstration on full-scale HPFF systems: Collaborate with a utility company to conduct field demonstration tests on a full-scale HPFF system. This will help validate the scalability of our findings and identify any performance discrepancies between controlled laboratory conditions and real-world scenarios.
2. Comprehensive attenuation model: Investigate the effects of varying backfill types, moisture levels, and pipe sizes on acoustic propagation to develop a comprehensive attenuation model that accurately reflects real-world environmental and operational conditions.
3. Advanced signal processing: Develop and implement advanced signal processing techniques to enhance pinpointing accuracy, particularly for signals that experience high attenuation or are affected by ambient noise and interference.
4. Integration with real-time monitoring systems: Explore the integration of the acoustic fault pinpointing method with real-time monitoring systems by deploying distributed sensors at key locations (e.g., every manhole) to enable real-time fault detection and pinpointing.

## Figures and Tables

**Table 1:** Detailed parameters of the test pipe.

Item	Value
Pipe length	19 m
Material	Carbon steel
Young’s modulus (E)	200 GPa
Density (ρ)	7850 kg/m^3^
Outer diameter (OD)	60.33 mm
Inner diameter (ID)	49.25 mm
Wall thickness	5.54 mm (Sch. 80)
External coating	Pritec
Internal coating	Epoxy

**Table 2:** Detailed parameters of the B&K accelerometers.

Item	Value
Product model	B&K 4518-002
Sensitivity	10 ± 10% mV/g
Measurement range	±500 g
Resonant frequency	62 kHz
Frequency range	1–20,000 Hz
Residual noise level	2000 µg
Maximum operational level (peak)	500 g
Mounting	Adhesive

Note: g is the acceleration of gravity.

**Table 3:** Detailed parameters of the B&K CCLD signal conditioner.

Item	Value
Product model	B&K 1704-A-002
Maximum frequency	55 kHz
Minimum frequency	2.2 Hz
Maximum gain (dB)	×100 (40 dB)
Minimum gain (dB)	×1 (0 dB)

**Table 4:** Specifications of the Delphin Expert Transient Data Logger.

Item	Value
Product model	Delphin Expert Transient
Number of input channels	4
Number of output channels	8
Voltage range	±25 V
Measurement accuracy	0.5 mV + 0.008%
Max. input frequency/min. pulse width	1 MHz/500 ns
Sampling rate	20–50 kHz

**Table 5:** Specifications of the Megger 32 kV Thumper.

Item	Value
Product model	Megger SPG 32
Voltage	0–32 kV
Energy	1750 Joules
Surge rate	3–10 s; single pulse
Burning	0–32 kV; 160 mA

**Table 6:** Summary of the experimental tests conducted.

Test	Objective	Pulse Generation	Embedment Condition	Filter
1.1	Acoustic velocity	Weight drop, pipe end	In air	20 kHz LPF
1.2	Localization accuracy	Weight drop, 1/3 length	In air	20 kHz LPF
2	Thumping-induced pulse	Fault Simulator, pipe end	In air	20 kHz LPF
3.1	Attenuation in air	Weight drop, 5 reps, pipe end	In air	3 kHz LPF
3.2	Attenuation on backfill	Weight drop, 5 reps, pipe end	On Tamped rock dust	3 kHz LPF
3.3	Attenuation embedded	Weight drop, 5 reps, pipe end	Fully embedded	3 kHz LPF
4	Fault pinpointing	Fault simulator, 1/3 length	Fully embedded	3 kHz LPF

**Table 7:** Peak amplitudes at each of the four locations (Test 3.1).

Title 1 Run#	Sensor 1 Peak (mV)	Sensor 2 Peak (mV)	Sensor 3 Peak (mV)	Sensor 4 Peak (mV)
1	1333	618.9	438.3	308.5
2	1481.5	714.5	505.3	346.8
3	1493.3	720	511.9	353.6
4	1582.5	778	551.9	379.8
5	1511	729.1	521.1	351.4
Average	1480.3	712.1	505.7	348.0
% Decrease		51.9%	29%	31.1%
Atten. Coefficient		0.116 Np/m	0.054 Np/m	0.059 Np/m

**Table 8:** Peak amplitudes at each of the four locations (Test 3.2).

Title 1 Run#	Sensor 1 Peak (mV)	Sensor 2 Peak (mV)	Sensor 2 Peak (mV)	Sensor 2 Peak (mV)
1	2114	892	586	365.3
2	1846	852.6	550.5	344.1
3	1961	909.6	590.8	362
4	2079	848.6	553.1	339.3
5	2070	855	551.5	338.2
Average	2014	871.6	566.4	349.8
% Decrease		56.7%	35%	38.2%
Atten. Coefficient		0.132 Np/m	0.068 Np/m	0.076 Np/m

**Table 9:** Peak amplitudes at each of the four sensor locations (Test 3.3).

Title 1 Run#	Sensor 1 Peak (mV)	Sensor 2 Peak (mV)	Sensor 2 Peak (mV)	Sensor 2 Peak (mV)
1	1716	233	13.4	4.4
2	1630	228	12.5	4.4
3	1663	233.2	13.2	4.4
4	1683	242.2	14	4.7
5	1662	234.6	13.3	4.1
Average	1670	234.2	13.3	4.4
% Decrease		86.0%	94.3%	66.9%
Atten. Coefficient		0.310 Np/m	0.453 Np/m	0.174 Np/m

## Data Availability

The data are contained within the article.
